# Predation of endangered Arctic foxes by Golden eagles: What do we know?

**DOI:** 10.1002/ece3.9864

**Published:** 2023-03-15

**Authors:** Craig R. Jackson, Lars Rød‐Eriksen, Jenny Mattisson, Øystein Flagstad, Arild Landa, Andrea L. Miller, Nina E. Eide, Kristine Roaldsnes Ulvund

**Affiliations:** ^1^ Department of Terrestrial Ecology Norwegian Institute for Nature Research (NINA) Trondheim Norway; ^2^ Norwegian Institute for Nature Research (NINA) Bergen Norway; ^3^ Department of Forestry and Wildlife Management, Faculty of Applied Ecology Agricultural Sciences and Biotechnology Inland Norway University of Applied Sciences Koppang Norway

**Keywords:** avian predators, carnivores, interspecific competition, intraguild predation, raptors

## Abstract

Dedicated conservation efforts spanning the past two decades have saved the Fennoscandian Arctic fox (*Vulpes lagopus*) population from local extinction, and extensive resources continue to be invested in the species' conservation and management. Although increasing, populations remain isolated, small and are not yet viable in the longer term. An understanding of causes of mortality are consequently important to optimize ongoing conservation actions. Golden eagles (*Aquila chrysaetos*) are a predator of Arctic foxes, yet little information on this interaction is available in the literature. We document and detail six confirmed cases of Golden eagle depredation of Arctic foxes at the Norwegian captive breeding facility (2019–2022), where foxes are housed in large open‐air enclosures in the species' natural habitat. Here, timely detection of missing/dead foxes was challenging, and new insights have been gained following recently improved enclosure monitoring. Golden eagle predation peaked during the winter months, with no cases reported from June to November. This finding contrasts with that which is reported from the field, both for Arctic and other fox species, where eagle depredation peaked at dens with young (summer). While the seasonality of depredation may be ecosystem specific, documented cases from the field may be biased by higher survey efforts associated with the monitoring of reproductive success during the summer. Both white and blue color morphs were housed at the breeding station, yet only white foxes were preyed upon, and mortality was male biased. Mitigation measures and their effectiveness implemented at the facility are presented. Findings are discussed in the broader Arctic fox population ecology and conservation context.

## Graphical abstract

Golden eagles represent a major threat to populations of endangered Arctic foxes in Fennoscandia, yet extremely little information has been published on the interspecific interactions. Here, we detail six confirmed depredation events that occurred at Norway's Arctic Fox Captive Breeding Station and relate findings to wild Arctic fox populations.

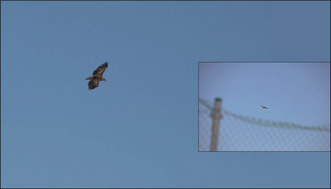

## 1 INTRODUCTION

Carnivores are a highly interactive group, and small‐ to medium‐sized carnivores are frequently exposed to strong top‐down effects which can influence the success of conservation initiates for threatened taxa (Vogel et al., 2019). The relative density of different carnivore guild members can have large effects on others. In terrestrial ecosystems, effects such as intraguild killing/predation, landscape of fear, kleptoparasitism, interference competition, and mesopredator release are well documented within mammalian carnivore guilds (Ritchie & Johnson, 2009). What is less well documented are lethal attacks and predation by top avian predators on mammalian carnivores (e.g., Moehrenschlager et al. (2007), Clark Jr (2009), Cypher et al. (2019)).

For the past 20 years, the endangered Fennoscandian Arctic fox population has received considerable conservation attention to save it from local extinction (Angerbjörn et al., 2013; Hemphill et al., 2020; Ims et al., 2017). Central to these conservation efforts, a captive breeding program was established in 2005 (Landa et al., 2017). Each year, captive‐born offspring are released into the wild, which has resulted in the successful re‐establishment of three locally extinct populations, as well as increasing the numbers in several other Norwegian populations (Hemphill et al., 2020; Landa et al., 2017, 2022).

Arctic foxes are small carnivores (ca. 3–4 kg; Audet et al. (2002)), and the Fennoscandian population is vulnerable to competition and predation from larger carnivores, such as wolverines (*Gulo*, 10–14 kg) and red foxes (*Vulpes*, 3–8 kg) (Frafjord et al., 1989, Tannerfeldt et al., 2002, Stoessel et al., 2019). Golden eagles are another protected species that are closely monitored across Norway (Gjershaug et al., 2018, Tovmo & Mattisson, 2021) and are reported as a natural predator of Arctic foxes across much of their distribution. While the effects of competition with, and depredation by red fox are well documented (Frafjord et al., 1989, Tannerfeldt et al., 2002, Pamperin et al., 2006, Rød‐Eriksen et al., 2023), there is a dearth of information on the interspecific interactions between Golden eagles and Arctic fox, and how this may affect the endangered canid's mortality rates. Camera trap studies reveal, however, that Arctic foxes display strong avoidance behavior at carcasses visited by Golden eagles (Rød‐Eriksen et al., 2023).

Elsewhere, and besides Arctic foxes, Golden eagles have been reported to kill Channel Island gray foxes (*Urocyon littoralis*, Roemer and Collins (2020)), swift foxes (*Vulpes velox*, Moehrenschlager et al. (2007)), and San Joaquin kit foxes (*Vulpes macrotis mutica*, Cypher et al. (2019)). Golden eagle predation drove the Channel Island fox population to the brink of extinction (Roemer & Collins, 2020), highlighting the significant effect these raptors can have on small carnivore populations.

Despite Golden eagles being routinely listed as a threat and predator in studies of the Arctic fox in Fennoscandia (Meijer et al., 2011, Rød‐Eriksen et al., 2023), there are no papers, to our knowledge, that specifically address the dynamics of this intraguild predation. The low number of records of direct interactions and predation events is most likely because Golden eagle predation of Arctic foxes occurs predominantly in remote wilderness areas, in the near absence of humans. Records of predation are thus rare and may be isolated incidents, often not formally communicated/published, that are fortuitously captured on wildlife camera traps (see for example Ims and Ehrich (2021)). As a consequence, documented cases typically lack detailed information about predation events and are incidences briefly mentioned within the context of general population ecology or monitoring (e.g., Ims & Ehrich, 2021; Ulvund et al., 2016). In such documentation, there is seldom more information than “killed by golden eagle” (e.g., (Johnsen, 2006, Landa et al., 2017, Ulvund et al., 2016).

Norway's Arctic fox captive breeding station makes use of large, open‐air enclosures located in the species' natural habitat. Here, we report on eight mortality events between 2019 and 2022; Golden eagle depredation was confirmed in six of these instances, while a lack of carcasses and/or images prevented confirmation of the other two potential eagle depredation cases. We detail characteristics of these depredation events as well as the effectiveness of mitigation measures at the captive breeding station. We discuss the interspecific predation patterns in light of ongoing conservation actions and implications for the wild Arctic fox populations.

## 2 BACKGROUND

### 2.1 The Arctic fox captive breeding station

**FIGURE 1:**
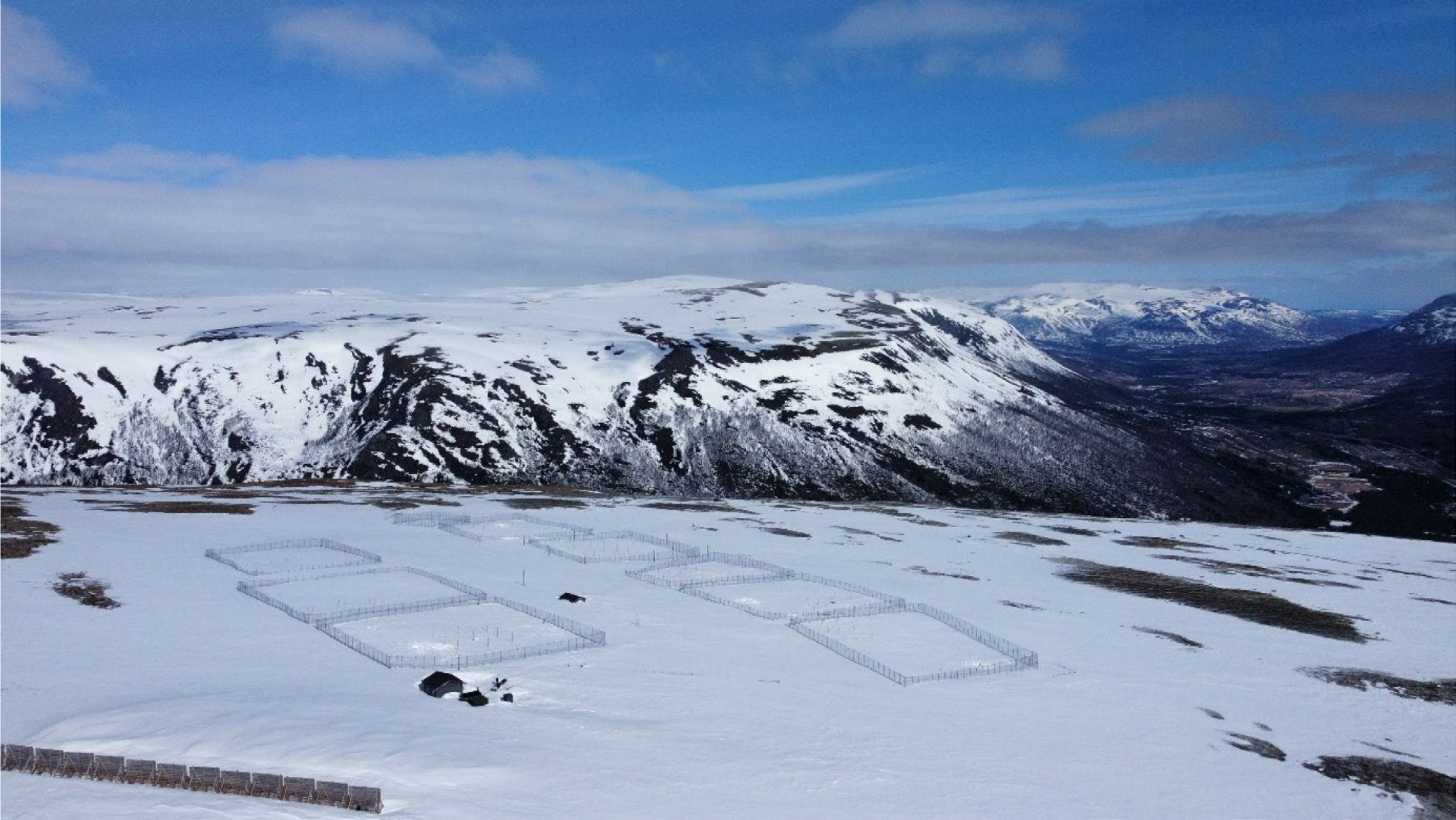
An eagle's eye view of the Arctic fox captive breeding station (April 2022).

The captive breeding station was constructed at Sæterfjellet, Oppdal Municipality, Norway, in 2005 (9°31.549 E, 62°27.230 N) (Landa et al., 2017). The station is located at 1280 m above sea level and in an area in which Arctic foxes naturally occur. The station consists of eight large enclosures (ca. 50 × 50 m) with one additional (smaller) enclosure which is used as a temporary holding enclosure when needed (Figure 1). Fences are 4.5 m in height. A breeding pair is kept in each of the main enclosures and their offspring are subsequently released into the wild. In addition to the prominent white morph, Arctic foxes commonly occur as a dark brown (“blue”) morph, and both these morphs are represented at the breeding station. There are no staff permanently based at the station, but a caretaker travels to the station every day to feed and check on the foxes and facilities.

The captive breeding station was located at the edge of a former Arctic fox sub‐population that went extinct in the late 1990s (Ulvund et al., 2016). Foxes released during the initial years of the breeding program (2007–2010) resulted in this population being successfully re‐established (Landa et al., 2017). Historical den site locations are consequently again in use by free‐ranging foxes, with 20 dens located within a 10 km radius of the breeding station.

Mating occurs from March to April with most pups being born between mid‐May and mid‐June. When the pups are ca. 10 weeks old, they are trapped, receive parasite medication, are ear tagged, and all relevant biological and demographic data are gathered. To minimize handling and interactions with humans, the foxes are not trapped or handled again before they are trapped for release in January. In mid‐January, the previous year's offspring are trapped and transported to a temporary holding facility. Once all juveniles (7–8 months old) are trapped, they are transported and released at predetermined release locations. From late January, only the adult breeding pairs are present at the breeding station. While the loss of juveniles to eagles prior to release reduces the number that can be released into the wild, the loss of adults to eagles has a potentially large impact on pup production at the captive breeding station. The loss of breeding females eliminates any chance of reproduction, whereas if males are killed post‐mating, the possibility for successful pup production remains.

Following the loss of three foxes during winter 2020/2021, an attempt to improve the monitoring of foxes and accurately ascertain causes of mortality was made. To this end, wide‐angled camera traps (model 5310WA, Ltl Acorn, Des Moines, Iowa, USA) were installed. Cameras were placed in the corner of each enclosure, with a wide field of view covering most of the enclosure, and units were programmed to take a photo every 10 min. A 10‐min time interval was reasoned as sufficient to detect eagles feeding on a fox (Hamel et al., 2013; Kays et al., 2020).

### 2.2 Golden eagle presence in the vicinity of the breeding station

**FIGURE 2:**
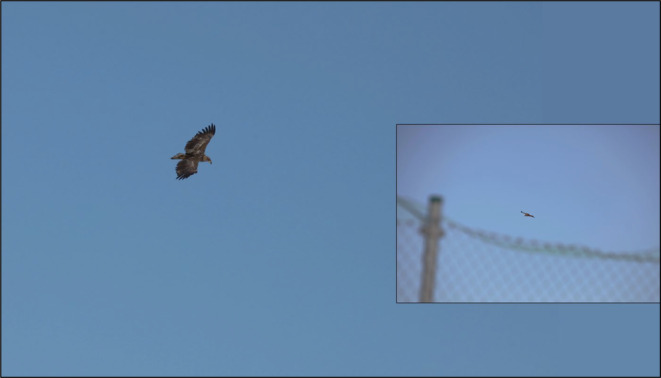
Golden eagle flying over the captive breeding station, April 2020.

Golden eagles are monitored both extensively and intensively across Norway. The population is stable and estimated at ~1000 occupied territories (or pairs) across the country (Mattisson et al., 2020). The intensive monitoring is conducted at 12 monitoring sites where 15 territories are monitored annually, and production of nestlings is documented. The captive breeding station is located within one of these sites, providing knowledge of the local eagle population. These eagles do not only represent a threat to the station foxes but likely also to the surrounding free‐ranging population (Figure 2).

Golden eagles are both a predator and a scavenger and are considered a generalist, feeding on a wide range of prey. The species prefers hunting in open terrain (Norberg et al., 2006; Watson, 2010) and uses its talons actively in the attack and killing of its prey. Based on prey remains from nests in Norway, the most common prey species are Ptarmigans (*Lagopus* sp.), mountain hares (*Lepus timidus*), forest grouse (*Galliformes* sp.), rodents, as well as semi‐domestic reindeer (*Rangifer tarandus*) and domestic sheep (*Ovis aries*), while red fox is also present to a varied degree (Jacobsen et al., 2022; Johnsen et al., 2007; Mabille et al., 2015; Norberg et al., 2006; Nybakk et al., 1999). It is often impossible to determine whether the remains of reindeer and sheep in the nest are killed or scavenged by the eagle, but it is well documented that the eagle can kill ungulates, especially small calves and lambs (Mabille et al., 2015; Norberg et al., 2006; Nybakk et al., 1999).

The lifting capacity of the Golden eagle, that is, how large prey the eagle can fly off with, depends on the eagle's body mass, wind conditions, and topography. Under normal conditions, maximum lifting capacity will be approximately half the eagle's own body mass (Watson, 2010). Eagles have been observed parting larger prey before bringing it to the nest (Watson, 2010). The body mass of the Golden eagle is approximately 3.5–5 kg, similar to that of an adult Artic fox, and therefore, an eagle will not normally be able to fly off with an adult fox out of the enclosures.

## 3 RESULTS AND DISCUSSION

### 3.1 Depredation of Arctic foxes at the captive breeding station

Between December 2019 and December 2021, a total of eight Artic foxes were lost; six of these were confirmed to have been killed by Golden eagles, while the remaining two are suspected but not verified. Monitoring and accounting for the daily presence of all Arctic foxes in the large enclosures is challenging, especially during winter, and many mortalities and/or disappearances were not immediately detected. After installing wide‐angle camera traps in April 2021 (details provided above), a total of four foxes were lost from the breeding station between May and December 2021. Only two of the carcasses were recovered, while the other two foxes were not present during the annual trapping in January. Checking the time‐lapse images confirmed that all four individuals were killed by Golden eagles (case‐specific details provided in Appendix 1).

Immediately prior to the installation of time‐lapse cameras, three foxes were lost during a 3‐month period. Only one of these carcasses was recovered, and although little remained, puncture wounds consistent with eagle talons were observed in the back‐shoulder region (see Appendix 1 for details). Captive adult and juvenile mortality, due to other causes, is extremely low. Based on the confirmed cases from 2020 to 2021, we believe that it is therefore probable that the two other foxes may also have been killed by eagles, yet this cannot be confirmed due to a lack of images/carcasses.

Only two confirmed cases of Golden eagle depredation were recorded during the first 10 years that the breeding station was operational (confirmed cases in 2012 and 2014; Landa et al. (2017)). However, during this same period (2006–2015), a total of 12 Arctic foxes, 6 adults and 6 juveniles, were either found dead (with an unknown cause of mortality) or never found (this excludes foxes known to have escaped, and pups that died prior to marking; info sourced from the captive breeding program's annual reports, 2006–2015). The confirmed depredation, therefore, provides retrospective insights into what may have occurred with individuals that were reported dead or missing, but with unknown causes. Despite this, the depredation rate during the presently described study period (2019–2021) was considerably higher than earlier (2006–2015). It is consequently possible that one or more of the long‐lived resident eagles had become accustomed to preying upon the foxes at the breeding station. However, as the eagles were not marked and picture quality low, we were unable to confirm if these eagles were indeed the same individual.

Furthermore, earlier eagle depredation events (pre‐time‐lapse cameras), although limited in number, could not specifically confirm that the foxes fed on by the eagles were actively hunted; although unlikely, the possibility exists that they died of other causes and that the eagles thereafter scavenged on their carcasses. The time‐lapse cameras both facilitated the documentation of what happened to dead/missing foxes and moreover confirmed that the foxes were alive immediately prior (<10 min) to being seen fed upon by eagles.

### 3.2 Characteristics of depredation events

In addition to the seven foxes (five confirmed killed by eagles) that were lost between December 2020 and December 2021, two eagles were observed feeding on a fox on the live web camera in December 2019 (see Appendix 1 for details). Of these eight losses (two unconfirmed causes of death), five occurred in December, one in December/January (exact date unknown), one in February/March (exact date unknown), and one in early May. Depredation was only evident during the winter months, with a peak in December.

The increased predation pressure observed during the winter months at the breeding station is most likely attributable to reduced food availability for the eagles. Carrion is an important food source for eagles during winter (Gjershaug et al., 2018), suggesting higher food stress during the winter months. Food stress may, therefore, explain the depredation of Arctic foxes during the winter months; with several eagle territories in close proximity to the breeding station, it is also likely that eagles become habituated to the presence of the foxes; their presence at the captive breeding station is rather predictable. In addition, foxes are likely to be more conspicuous and exposed to the snow compared to the summer. Furthermore, observations of eagles near the breeding station are rare during summer, yet common during the winter months (Pers. obs.), suggesting altered ranging/foraging behavior.

Our finding of a distinct depredation peak in winter contrasts with anecdotal reports of eagle depredation in the wild, which almost exclusively entail reports of depredation during summer (e.g., Ims & Ehrich, 2021; Meijer et al., 2011). These discrepancies could most likely be explained by a lack of longer‐term monitoring at den sites during winter. During winter, short visits are made to assess activity at the den sites (Ulvund et al., 2016), while during summer more intensive observations, increasingly aided by the deployment of camera traps, are performed to assess reproductive activity and litter size. Depredation of captive foxes during winter implies that wild Arctic foxes are also vulnerable to increased depredation during winter, although this is extremely difficult to document in the wild given their low densities and occurrence in remote areas. Increasing use of wildlife camera traps to monitor the Arctic fox population in winter could, however, shed new light on the interactions and potential conflicts with Golden eagles.

**FIGURE 3:**
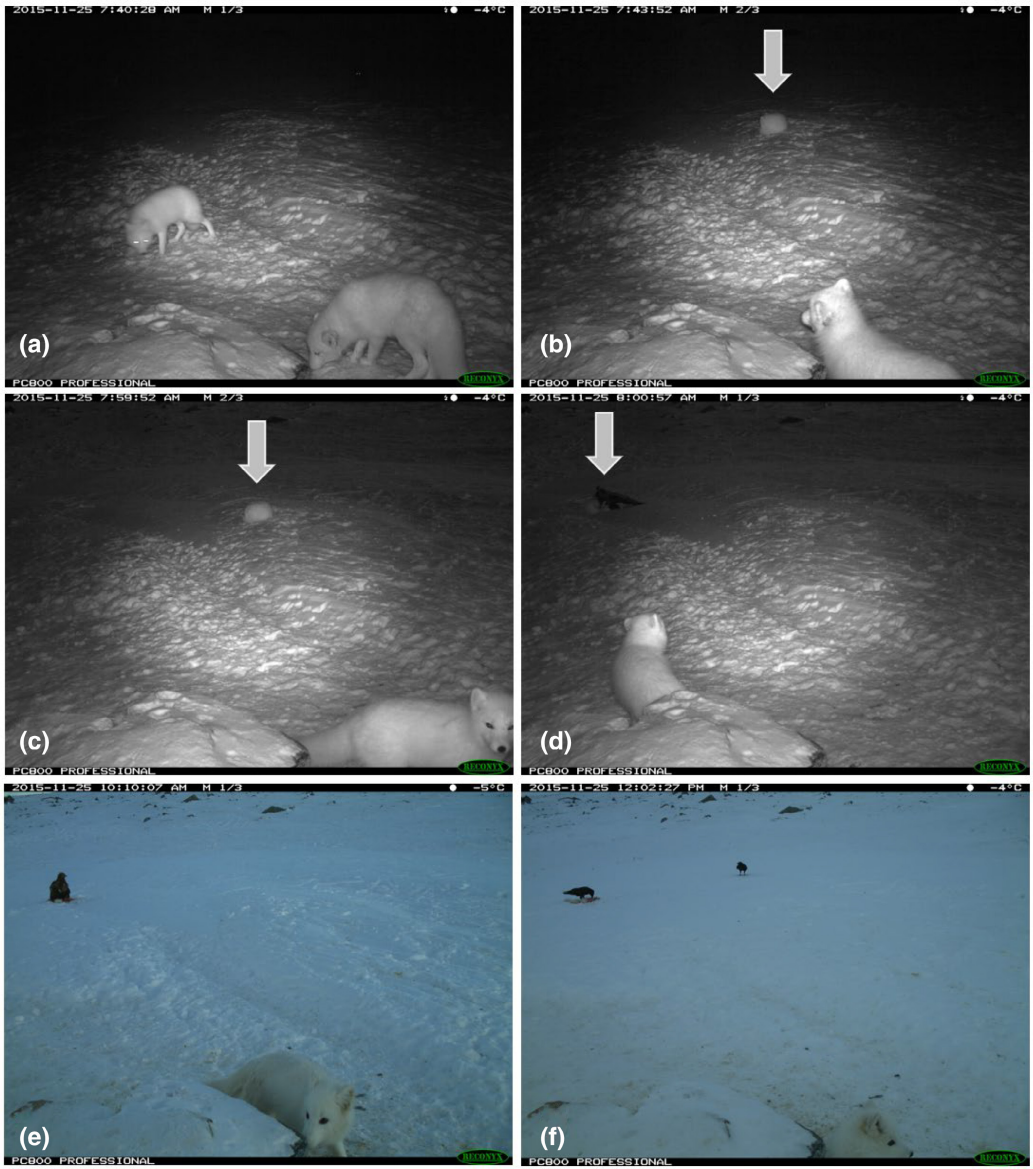
Selected images summarize the sequence of events where an Arctic fox was killed outside a feeding station, on November 25, 2015. Two individuals were seen outside the feeding station (a), and shortly thereafter, one of them lay down, seemingly asleep (b). Sixteen minutes later, at 7:59 a.m., this individual had not moved (c) and at 8:00 a.m. an eagle was in the process of killing the fox (d). The eagle and other fox remained on site for over 2 h (e), and later a pair of ravens arrived and scavenged the remains (f).

As part of the ongoing conservation efforts, more than 250 feeding stations have been deployed across the species distribution in Fennoscandia. Feeding stations are often placed in close proximity to Arctic fox dens and camera traps are installed to record activity of Arctic foxes and other species visiting the feeding stations. Although these cameras are positioned to focus on the area immediately in front of feeding stations (to facilitate identification of Arctic fox ear tags) and thus have a restricted field of view, Golden eagle depredation has been fortuitously recorded in a couple of instances. In early winter 2015, two foxes were first seen outside a feeding station in Sylan, Central Norway (Ulvund et al., 2016). After initially moving about (Figure 3a), the one fox curled up and seemingly went to sleep at 7:43 am (Figure 3b). At 7:59 am, this individual was still in the same position (Figure 3c). The next image, taken at 8:00 am, shows an eagle sitting on top of this fox (Figure 3d,e). Apart from confirming that foxes are vulnerable to depredation during winter in the wild, there are many similarities with depredation events recorded at the breeding station. Firstly, the event occurred in early winter and the killed individual was curled up sleeping, with no signs of vigilance behavior (see below). Furthermore, a pair of ravens appeared after the eagle had made the kill (Figure 3f), something also observed at the breeding station (often several birds eventually arrived; Appendix 1). Interestingly, the other fox remained present during this entire series of events, totaling more than 4 h.

In contrast to red foxes, Arctic foxes have not been detected in diet studies of Scandinavian Golden eagles (Hoegstroem & Wiss, 1992; Johnsen et al., 2007; Norberg et al., 2006; Nybakk et al., 1999; Nyström et al., 2006; Tjernberg, 1981), suggesting that they are not regular prey items for breeding eagles during summer. This could maybe partly be explained by low spatial overlap between these studies and Arctic fox presence. However, no diet studies have been conducted during winter, which was when predation occurred at the breeding station and was also recorded in the wild. Furthermore, Arctic fox population sizes have been very low during recent decades, making the detection of potential fox remains highly unlikely.

### 3.3 Characteristics of depredated foxes

Of the six confirmed cases reported here, five were males while the sex of the sixth individual was unknown (30 of 31 pups were successfully trapped and marked during July and August 2021; the only unmarked individual was killed in December 2021). The two individuals that were lost during the same period, but not confirmed as killed by eagles, included one breeding female and one juvenile male. Predation events were, therefore, strongly male biased.

Observations of fox behavior at the station suggest that during the annual reproductive period (March–May), males are more likely to lie outside the den entrance while females spend more time inside the den (Pers. obs., the authors). During this period, the breeding males' apparent mate‐guarding strategy may result in them being more exposed and vulnerable to eagle depredation.

All confirmed depredation events (as well as the two that died/disappeared due to unknown reasons) were white color morphs, despite both the white and blue color morphs being represented at the captive breeding station. The proportion of each color morph differs between years, but in 2021, for example, 21% (9 of 45) of all foxes were blue. Of the 12 foxes that disappeared/died due to unknown reasons between 2006 and 2015, only one was blue; he disappeared and was never found.

Although the captive breeding program has only released approximately 10% blue foxes, this color morph appears to have greater fitness and the proportion of blue in the re‐established populations have increased to ca. 25% (Di Bernardi et al., 2021). Although the sample sizes were small and can in no manner be used to infer greater predator avoidance abilities by blue Arctic foxes, the trend is noteworthy given the pronounced color‐specific fitness differences reported in the wild (Di Bernardi et al., 2021).

### 3.4 Post mortem findings – How are foxes killed?

Eagles are dependent on their powerful talons to capture and kill prey. We did not observe puncture wounds in the skull, as is often seen when Golden eagles kill ungulate prey species (Skåtan & Lorentzen, 2011) or as reported in depredation on other fox species in other ecosystems (Cypher et al., 2019). Instead, puncture wounds were evident across the dorsal shoulder–neck region of the Arctic foxes. This may be because the foxes are fairly small, and the lungs are punctured effectively in this way.

**FIGURE A7:**
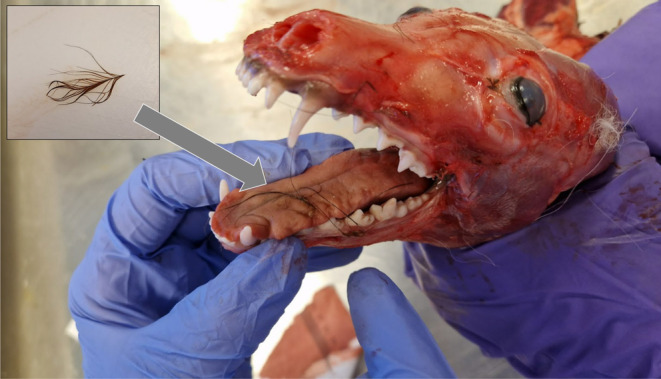
Upon closer inspection during the post mortem, feathers were found in the fox's mouth. The fox's mouth was closed when recovered shortly after death, and it is therefore presumed that the fox bit at the eagle when it was attacked. The same was found in Case 8, reported hereunder.

In two of the post mortems, feathers were found inside the foxes' mouths (see Figure A7, Appendix 1, cases 5 and 8). It, therefore, appears as though these individuals attempted to defend themselves when attacked and managed to bite at the eagles.

### 3.5 Mitigation measures at the captive breeding station

The release of captive‐bred animals into the wild has frequently resulted in high mortality rates due to poor and underdeveloped antipredator behavior (Jule et al., 2008). Consequently, natural exposure to the eagles can be viewed positively as this may reduce post‐release mortality rates and improve the likelihood that conservation goals are met. Yet the foxes' confined location within the enclosures and proximity to local Golden eagle territories result in them being particularly vulnerable to depredation. Thus, although the Arctic foxes are kept under semi‐natural conditions and Golden eagle predation is natural in the wild, there are important ethical and animal welfare considerations given that they are kept in captivity.

In addition, the loss of foxes to eagles (or any other source of mortality) directly impacts the captive breeding program achieving its conservation goals as the number of foxes that can be released into the wild is decreased. Therefore, to reduce depredation risks, a series of mitigation measures were implemented in 2021. These included the installation of (i) feeding boxes to reduce the presence and foraging of fox food by large flocks of corvids, (ii) rotating reflective bird deterrents, (iii) simple structures, and (iv) obstacles to inhibit aerial depredation events by Golden eagles. More information on each of these measures is provided in Appendix 1 while only the main findings are presented here.

**FIGURE A14:**
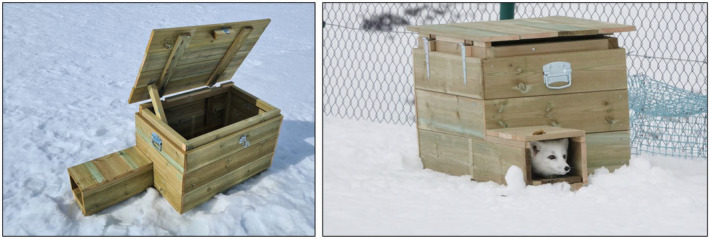
Wooden feeding boxes were installed in each enclosure in an attempt to make the foxes' food inaccessible to crows and ravens. This has functioned efficiently and drastically reduced the density of scavenging birds.

**FIGURE A15:**
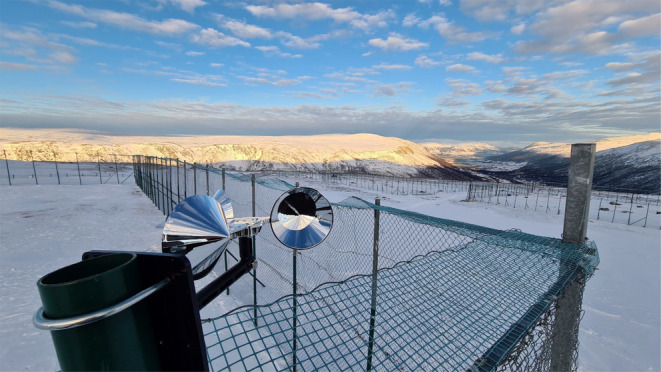
Rotating, light‐reflecting bird deterrents were installed in all enclosures but failed to have the desired effect.

Large flocks of crows and ravens, often numbering between 30 and 40 individuals, had become accustomed to scavenging on the food set out for the foxes. This situation was undesirable as the presence of crows and ravens could attract Golden eagles, the birds' persistent presence could desensitize foxes to aerial approaches by potential avian predators, ravens could kill young pups during the first few weeks after they emerge from the den (Chevallier et al., 2016), and the birds additionally consumed substantial volumes of fox food. In May 2021, wooden feeding boxes with tunnel entrances were built and placed in each enclosure (Figure A14, Appendix 1), which resulted in the disappearance of the birds. Approximately 6 months before the feeding boxes were taken into use, rotating, reflective bird deterrents were trialed (Figure A15, Appendix 1), but these failed to deter ravens, crows, or eagles.

**FIGURE A16:**
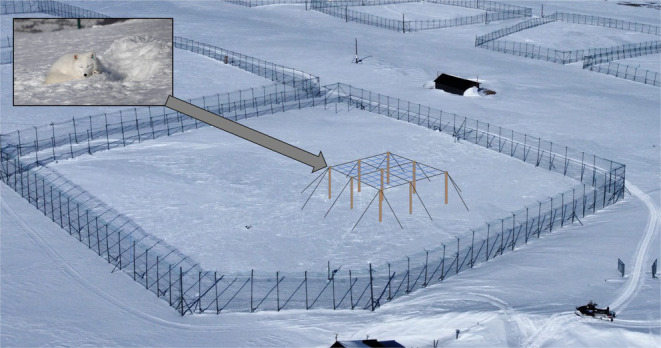
An illustration of the envisioned structures, located immediately above dens.

Images obtained from the time‐lapse cameras revealed that foxes were particularly vulnerable to eagle attacks when lying on the snow outside of the den entrance. A simple construction, consisting of tall wooden poles and a series of wire cables and ropes, spanning the area immediately above and around one den in each enclosure, was piloted in September 2021 (Figures A16, Appendix 1). In December 2021, a video surveillance camera captured the moment when a Golden eagle attempted to attack a pair of foxes in enclosure 1. The foxes were active and detected the eagle's rapid, targeted approach, upon which they fled at full speed toward the den. The eagle was forced to abort that attack at the last second, as the overhead ropes and cables were detected and thus avoided. This both showed that the simple structures could reduce depredation risk, as well as that the ropes were visible to the eagle and did not result in a collision and potential injuries.

Each enclosure has two to three dens and the pilot project only allowed for the construction of a single structure in each enclosure. Almost immediately after the abovementioned predation attempt, a fox was killed in enclosure 4 (as evidenced by time‐lapse camera images and associated timestamps; see Appendix 1). The image revealed that this fox was killed outside a secondary and unprotected den entrance, where one or more foxes had been seen lying during the preceding 2 h (based on time‐lapse images taken every 10 min). Indeed, of the three foxes killed within a 3‐week period in December 2021, at least two were outside of secondary dens. To reduce such risks associated with depredation outside of unprotected den entrances, bamboo sticks were purchased and erected in the snow around other den entrances. These obstacles make it difficult for a rapidly approaching eagle to swoop down and catch foxes. We have no direct observations or images that could be used to qualify the effectiveness of the bamboo sticks, but after deploying sticks in early January 2021, no foxes were lost. During early winter (November to mid‐December), snow depths are often fairly shallow, making it difficult to securely anchor the sticks in the snow. In the future, sticks will be deployed during autumn (holes drilled into the ground), thereby hopefully better protecting the foxes from the start of winter (implemented in October 2022; ca. 300 sticks erected).

### 3.6 Relevance to wild Artic foxes

Although predation on Arctic foxes is rarely documented in the wild, Golden eagles are frequently observed visiting den sites both during winter and summer (observations during den controls and pictures from camera traps, *unpublished data, Norwegian Arctic fox monitoring programme*), and a recent experimental study revealed that Arctic foxes avoided simulated carcasses in areas where Golden eagles were present (Rød‐Eriksen et al., 2023). Although the causal relationship was not explicitly established, Larm et al. (2020) suggest that higher pup survival at dens experiencing regular visits by tourists may be due to lower activity by Golden eagles.

The competitive interspecific interactions also highlight how the conservation management activities pertaining to two protected species may result in unforeseen challenges. Long‐term protection in Norway has resulted in a stable Golden eagle population for the past ca. 20 years (Mattisson et al., 2020; Tovmo & Mattisson, 2021). With as few as 40–60 adult Arctic foxes remaining in Fennoscandia during the early 2000s, however, the species' population size has gradually increased following two decades of concerted conservation efforts (Landa et al., 2017). Still, the eagles represent a real threat to the foxes and in certain areas, park rangers and local management authorities are concerned that the Golden eagle could limit the re‐establishment of endangered Arctic fox populations. Due to the eagles' protected status, the choices for mitigation efforts are limited and creativity is needed. In response to the threats from the protected eagles, reflective bird deterrents have recently been trialed in northern Sweden, although similar devices proved ineffective at the breeding station. In contrast, bamboo sticks have seemingly been more effective and may serve as a cheap, non‐invasive method that could too be trialed in the wild. Areas immediately surrounding dens or supplementary feeding stations may be targeted for such mitigation measures and reduce predation risk during both summer (adults and pups) and winter (adults).

## AUTHOR CONTRIBUTIONS

**Craig R. Jackson:** Conceptualization (lead); data curation (equal); investigation (equal); project administration (lead); writing – original draft (lead); writing – review and editing (lead). **Lars Rød‐Eriksen:** Conceptualization (equal); investigation (equal); methodology (equal); writing – original draft (equal); writing – review and editing (equal). **Jenny Mattisson:** Conceptualization (equal); formal analysis (equal); investigation (equal); methodology (equal); writing – original draft (equal). **Øystein Flagstad:** Writing – review and editing (equal). **Arild Landa:** Writing – review and editing (equal). **Andrea L. Miller:** Conceptualization (equal); data curation (equal); investigation (equal); writing – original draft (equal); writing – review and editing (equal). **Nina E. Eide:** Conceptualization (equal); investigation (equal); writing – original draft (equal); writing – review and editing (equal). **Kristine Roaldsnes Ulvund:** Conceptualization (lead); data curation (lead); formal analysis (equal); investigation (equal); methodology (equal); project administration (equal); writing – original draft (lead); writing – review and editing (lead).

## Figures and Tables

**FIGURE A1:**
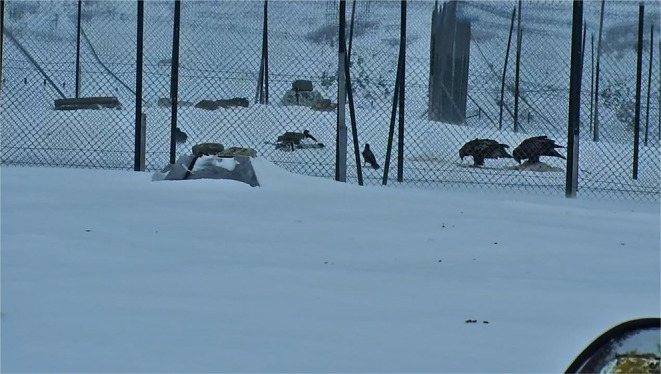
Two Golden eagles feeding on an Artic fox in enclosure 2, on December 2019. Screenshot of live‐video stream (Arctic fox captive breeding program/NINA).

**FIGURE A2:**
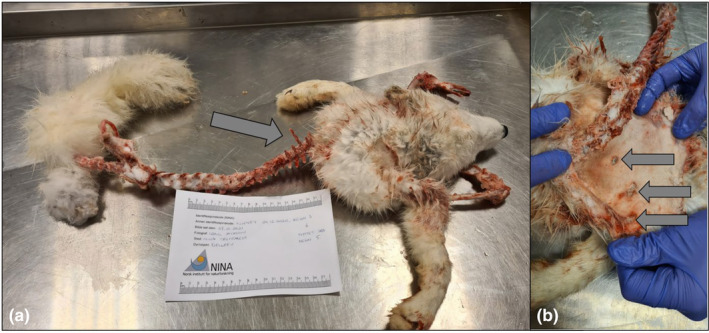
The remains of a juvenile fox killed in enclosure 3 in December 2020. Insert (a) shows that extremely little of the carcass remained. The severing of the rib bones is another diagnostic feature of Golden eagle feeding behavior (Skåtan & Lorentzen, 2011). Furthermore, insert (b) shows that puncture wounds were evident in the dorsal shoulder–neck region.

**FIGURE A3:**
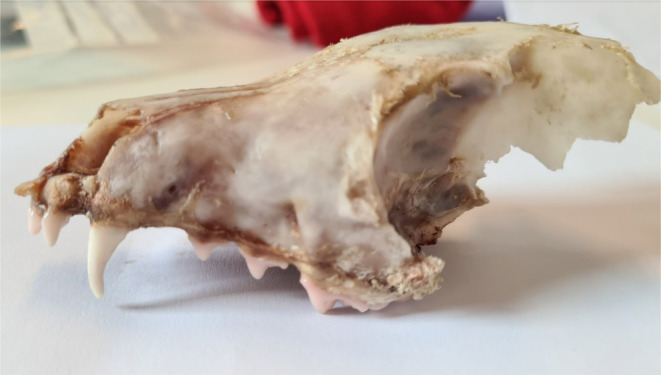
The remains of an Arctic fox found approximately 1 month after its death.

**FIGURE A4:**
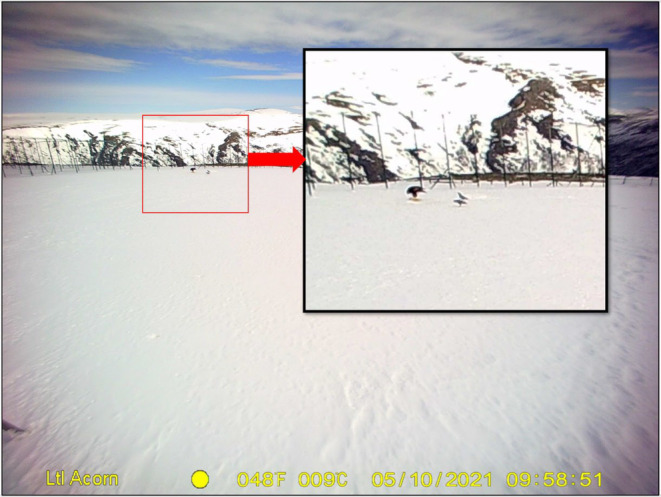
A time‐lapse image showing the eagle feeding on the fox, with two corvids waiting their turn. The first image of the eagle on the fox was taken at 9:28 a.m., while the image above was taken 30 min thereafter. Photo: Arctic fox captive breeding program/NINA.

**FIGURE A5:**
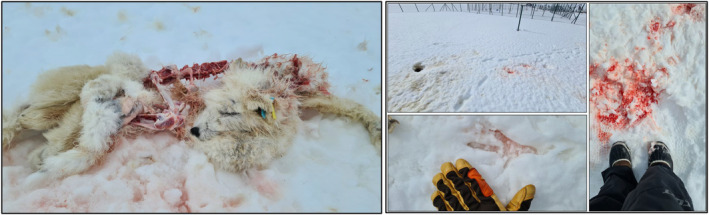
The adult breeding male as found 4 h after being killed by an eagle. He was caught immediately outside of the den entrance, which was both evidenced by inspection of the enclosure and the camera images.

**FIGURE A6:**
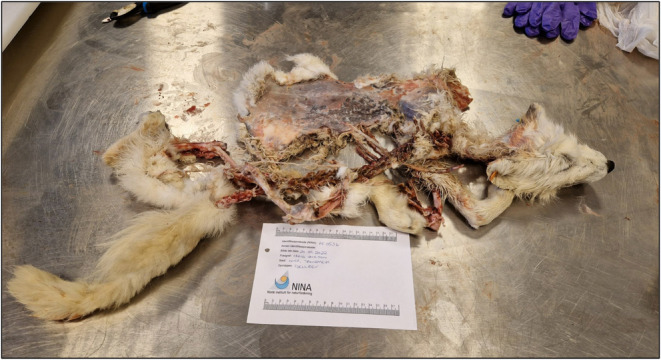
Adult male killed 4 h before the carcass was recovered, photographed here during post mortem investigations in the lab. Even after only a short time, little remained of the fox. Accurately determining the cause of death based on a post mortem of the remaining body parts alone would have been extremely challenging.

**FIGURE A8:**
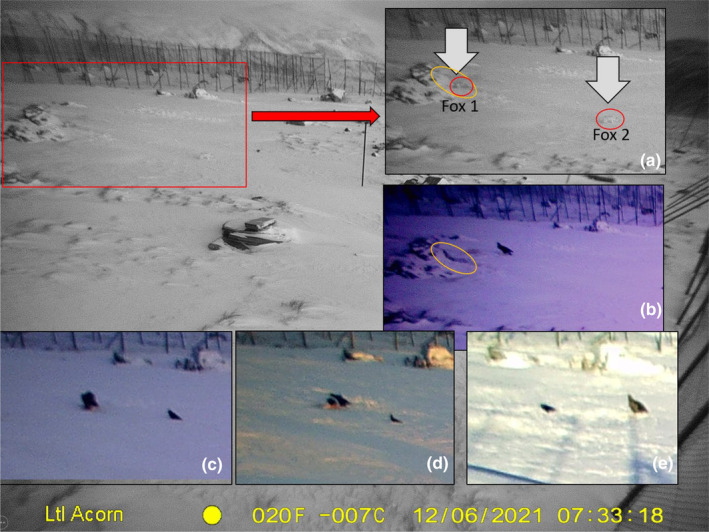
Insert (a) shows the position of both foxes (arrows and red ellipses) at 7:33 a.m. Insert (b) is at 7:44 a.m. and shows an eagle on the ground. The two orange ellipses in inserts (a) and (b) indicate displaced snow at and alongside where the fox was lying, which most likely occurred during the attack and ensuing struggle. Inserts (c) and (d) are extracts from some of the additional images showing the Golden eagle feeding on the adult breeding male in enclosure number 5. The last image of the eagle (insert e) was taken at 11:53 am. Photos: Arctic fox captive breeding program/NINA.

**FIGURE A9:**
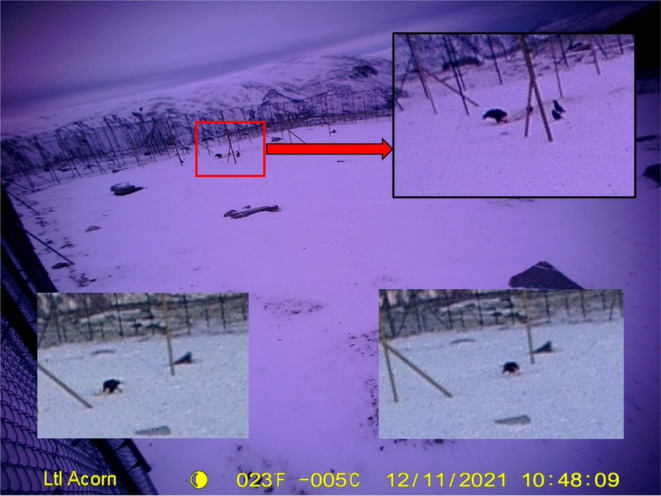
An eagle feeding on juvenile Arctic fox in enclosure 8. Note that the time on the image is set to Central European Summer Time, and hence, the actual time was 9:48 a.m. Photos: Arctic fox captive breeding program/NINA.

**FIGURE A10:**
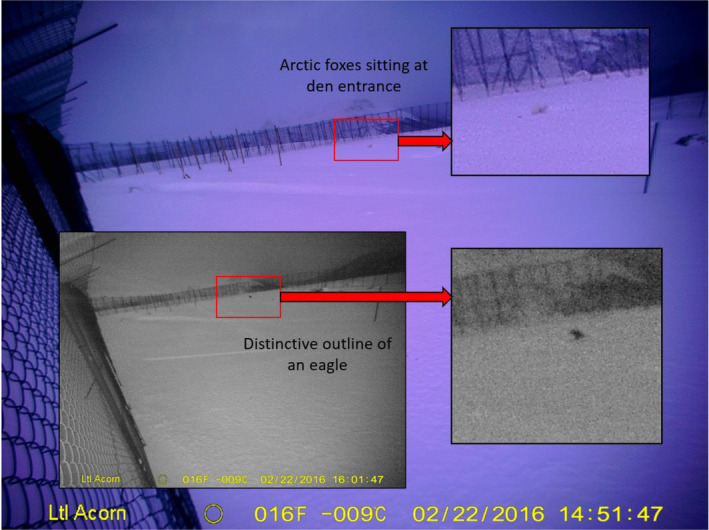
The pictures show two Arctic foxes outside a den entrance. Subsequent images reveal an eagle at this same location. Pictures were taken immediately before sunset (time stamp on image is correct and date is incorrect). The following morning, the fox's remains were recovered by the caretaker. Photos: Arctic fox captive breeding program/NINA.

**FIGURE A11:**
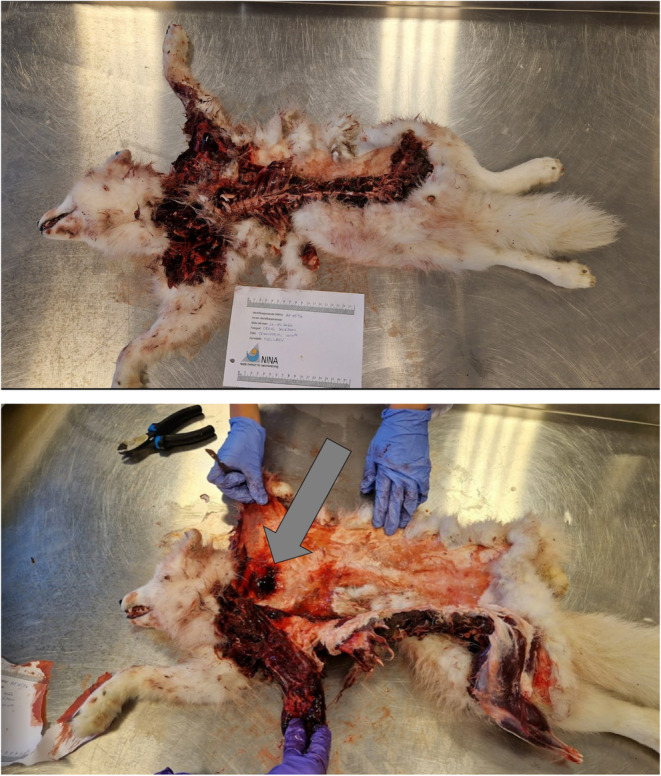
Upon skinning the fox, puncture and trauma wounds were evident along the shoulder and neck region. Photos: Arctic fox captive breeding program/NINA.

**FIGURE A12:**
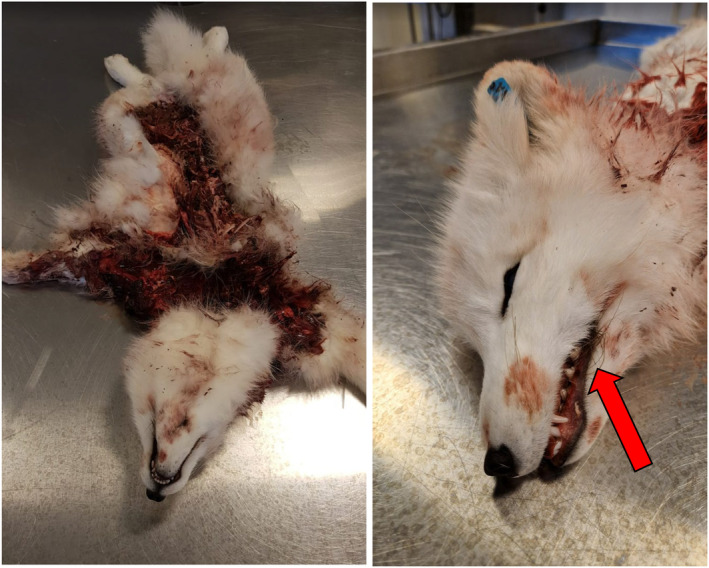
Feathers were also found in this fox's mouth (red arrow) and, as per Case 5 above, are presumably Golden eagle feathers. Photos: Arctic fox captive breeding program/NINA.

**FIGURE A13:**
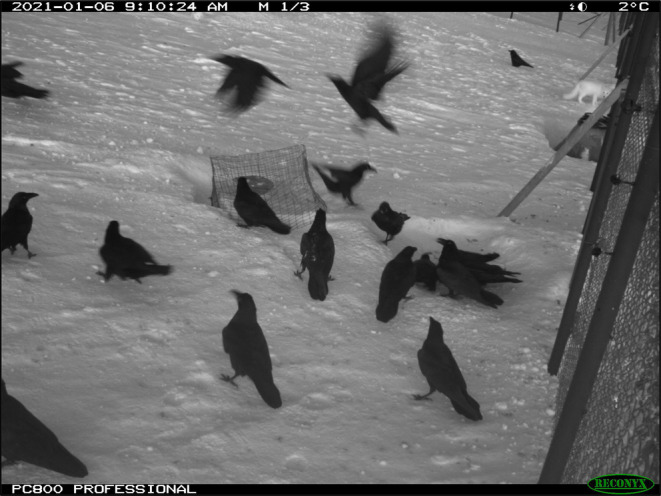
An example of the daily occurrence of large numbers of crows and ravens at the breeding station. Photo: Arctic fox captive breeding program/NINA.

**FIGURE A17:**
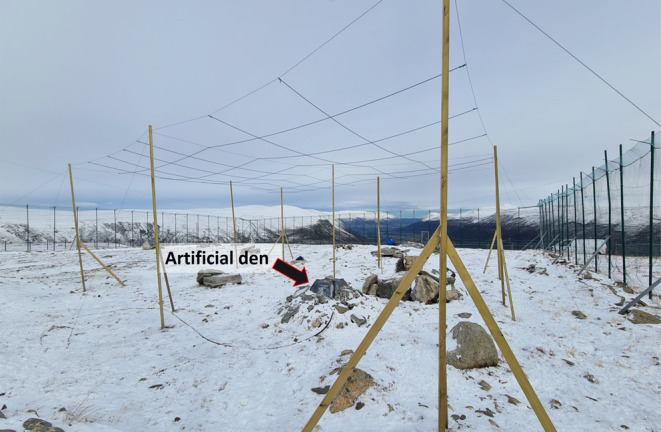
A simple construction positioned above an Arctic fox den in an attempt to reduce Golden eagle depredation risk.

**FIGURE A18:**
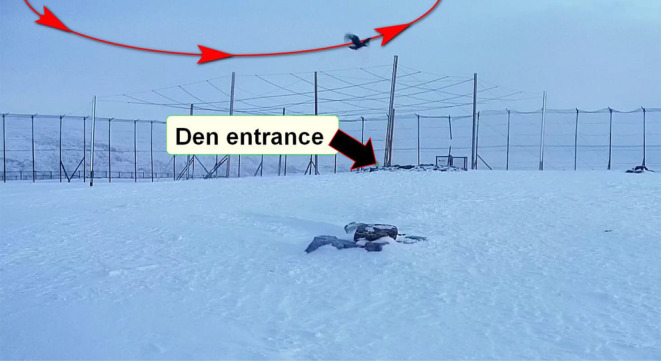
A screenshot taken from video footage that showed a Golden eagle attempting to catch an Arctic fox in enclosure 1. In the video, two foxes are seen sprinting toward the den entrance, at which point a Golden eagle appears, at speed, trying to catch them, but averted the chase when it got close to the ropes above the den. The eagle is visible in the image and the red line and arrows depict its approximate flight path. At this point in the video, the foxes are not visible.

**FIGURE A19:**
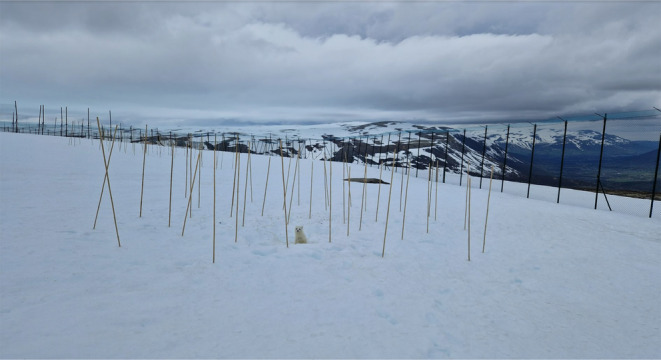
A fox emerging from its den entrance and surrounded by bamboo sticks intended to create an obstacle for approaching eagles.

## Data Availability

All data on which this manuscript is based are presented and included in Appendix 1.

## APPENDIX 1 Details pertaining to the loss of eight captive Arctic foxes between December 2019 and December 2021.

### A.1 CASE 1: December 2019 – Juvenile male, enclosure 2 (confirmed eagle depredation)

On December 19, 2019, between 10 and 10:30 a.m., observations on one of the breeding stations’ live‐streaming video cameras revealed two Golden eagles feeding on a fox (Figure A1) (Ulvund et al., 2020). The caretaker was immediately notified and traveled to the station shortly thereafter. Except for the head, backbone, skin, and legs, little remained for the post mortem investigation. There were no obvious signs of predation (such as talon marks), but the carcass had been picked clean by the eagles and scavenging corvids. With so little remaining, the post mortem alone could not determine cause of death. However, the healthy character of the remaining skin, fur, and muscles, as well as the presence of a good amount of subcutaneous fat over the back muscles/hips, suggested that the fox was relatively healthy (though examination of the organs would have been needed to confirm this). There was also very little post mortal change in the muscles (i.e., the carcass was fresh). Consequently, all evidence suggests that the fox was alive and killed by an eagle, rather than scavenged after dying from another cause.

### A.2 CASE 2: December 24, 2020 – Juvenile male, enclosure 3 (confirmed eagle depredation)

Remains were found in enclosure 3, but little of the carcass remained intact (Figure A2a). Despite this, puncture wounds consistent with Golden eagle predation were found in the dorsal shoulder–neck region (Figure A2b).

### A.3 CASE 3: December 2020/January 2021 – Juvenile male, enclosure 3 (potentially killed by eagle)

Three weeks after Case 2, in mid‐January 2021, trapping commenced in preparation for release. It then became apparent that another juvenile from the same enclosure was missing. The date of disappearance could not be established, but based on observations of all individuals it could be narrowed down to the December–January period. This fox was never found, so the cause of death/disappearance could not be ascertained.

### A.4 CASE 4: March 16, 2021 – Adult breeding female, enclosure 7 (potentially killed by eagle)

On March 16, 2021, a skull was found outside enclosure number 7. It was apparent that it was not fresh (Figure A3), and DNA analysis revealed that this was the breeding female from enclosure 7. Reviewing standard camera‐trap images from the enclosure (this occurred before the wide‐angle time‐lapse cameras were installed) showed that the last recorded image of her alive was almost 1 month prior to when she was found. Here again, it was not possible to determine when she died (narrowed down to February–March) or the reason for her death. Given that adult Arctic fox mortality within the enclosures is low, and the other confirmed eagle depredation cases occurred during the same time period, it is plausible that one or both (Cases 3 and 4) of these foxes may have been taken by eagles.

### A.5 CASE 5: May 10, 2021 – Adult breeding male, enclosure 8 (confirmed eagle depredation)

First photo of eagle on fox at 9:28 a.m.

The time‐lapse cameras were installed on April 27, 2021 and on May 10, 2021; only 13 days later, the first eagle depredation was documented when a freshly killed fox was found in enclosure 8. In this instance, eagle tracks were apparent in the snow. Reviewing images captured by the time‐lapse camera (Figure A4) showed that the fox was killed 4 h prior to it being discovered. The images also confirmed that both foxes were active and moving around shortly before being killed; the fox was therefore actively preyed upon. The male spent considerable time lying outside the den entrance, and this is where he was killed (Figure A5). This male had successfully mated, and the female subsequently raised eight pups by herself. Little remained (Figure A6), and feathers were found in the fox's mouth (Figure A7), presumably due to the fox trying to defend itself after being attacked.

### A.6 CASE 6: December 6, 2021 – Adult breeding male, enclosure 5 (confirmed eagle depredation)

First photo of eagle on fox at 7:43 a.m.

This was a new breeding pair, and there were therefore no juveniles in the enclosure. Foxes are not particularly active during December, especially during bad weather. However, after repeatedly only seeing a single individual, the memory card from the time‐lapse camera was checked. The images confirmed that both foxes were alive until the morning of December 6, 2021. The preceding image, taken 10 min before the eagle was seen, showed one fox walking around in the middle of the enclosure while the other was outside the den entrance (Figure A8, insert a). Numerous images of the eagle feeding on the fox were obtained, and the bird remained in the enclosure for 4 h. The carcass of this fox was not found, and no remains or ear tags were found when searching the enclosure following snow melt. In the absence of the images, this fox would have been reported as “missing.”

### A.7 CASE 7: December 11, 2021 – Juvenile, enclosure 8 – (confirmed eagle depredation)

First photo of eagle on fox at 9:35 a.m.

In January, the foxes are trapped in preparation for the transportation and release of the previous year's offspring. All foxes are trapped, including the adults, to ensure that the enclosure is empty. It is particularly challenging to account for all individuals during winter, and the trapping also facilitates the identification of foxes that are potentially missing. In January 2021, all foxes in enclosure 8 were trapped. After several days of leaving traps active, no signs of activity were found, yet one animal remained unaccounted for. Photos from the time‐lapse camera were downloaded and systematically assessed. The images revealed an eagle feeding on a fox (Figure A9). The fox was not found and would have been reported as “missing” in the absence of these images.

### A.8 CASE 8: December 26, 2021 – Juvenile male, enclosure 4 – (confirmed eagle depredation).

First photo of eagle on fox at 2:41 p.m.

The juvenile male was found dead at 9:15 a.m. on the 27th of December. The fox was found by the station caretaker during his daily rounds to feed the foxes. Looking at the images from the wildlife camera traps, it is clear that the fox was killed on December 26th, right before sunset. In the images, two foxes can be seen outside the den entrance. Half an hour later, an eagle can be seen sitting on the ground where the foxes were resting.

Interestingly, this depredation event occurred immediately after a failed hunt that involved an eagle and two foxes in enclosure 1 (Details below and in Figure A18). While the pair that managed to escape were saved by structures erected to minimize Golden eagle depredation risk, this individual was killed while lying in the open, with no protection from aerial predators (Figure A10).

Puncture wounds from the eagle's talons were clearly visible (Figure A11) and feathers were found in the fox's mouth (Figure A12).

### A.9 Mitigation measures.

To reduce depredation risks, a series of mitigation measures were implemented at the captive breeding station in 2021.

#### A.9.1 Feeding boxes

The foxes were fed with a nutritionally balanced food developed for the fox farming industry. Food was traditionally placed inside the enclosure, directly on the ground/stones or snow. A wire net was placed over the food to hinder crows and ravens. This, however, was not effective and over the years, the birds had become accustomed to the predictable food source. On a daily basis, mixed flocks of up to 40 corvids moved from enclosure to enclosure, scavenging the foxes' food (Figure A13).

This situation was undesirable as (1) the presence of crows and ravens could attract Golden eagles; (2) the birds' persistent presence could desensitize foxes to aerial approaches by potential avian predators; (3) ravens could kill young pups during the first few weeks after they emerge from the den (Chevallier et al., 2016); and (4) the birds consumed substantial volumes of the fox food.

During May–June 2021, feeding boxes were built and placed in each enclosure (Figure A14). The boxes were of a simple design and were constructed of wood. The tunnel entrance was hoped to exclude birds, and to date, we have not recorded a single bird entering the feeding boxes (additional, motion‐triggered camera traps are positioned to capture images of activity around feeding boxes). Soon after the feeding boxes were deployed, the crows and ravens disappeared. During early winter (November–December), some birds did return, but in very low numbers (mostly pairs). Instead of having direct access to food, they were forced to move about the enclosures and search for scraps.

### A.10 Rotating bird deterrents

In January 2021, rotating bird deterrents were placed in each enclosure, directly above the feeding sites (Figure A15). Despite the units rotating efficiently, they had little effect and did not deter crows, ravens, or eagles.

### A.11 Eagle deterrent structures.

Given harsh weather conditions at the breeding station, it is not feasible to cover entire enclosures with netting. This was previously attempted but icing, snow, and extreme winds proved too much for the nets. The mortalities captured on time‐lapse camera also illustrated that most foxes were killed in close proximity to a den entrance. As a consequence, we attempted to design smaller structures that had little wind resistance and fewer ropes, which were strategically placed above dens in an attempt to avert killing when foxes were lying outside den entrances (Figure A16).

As a pilot project, one such structure was constructed in each of the eight main enclosures in September 2021. Nine wooden planks, each measuring between 4.0 and 4.5 m in length, were used as the main uprights. These were anchored to the ground and each other using wire. Tough, weather‐resistant rope was then used to create additional obstacles to approaching eagles. An example of a completed structure is shown in Figure A17.

### A.12 Effectiveness of the eagle structures.

On December 26, 2021, at 2:31 p.m., exactly 10 min before the first image of the eagle on the fox reported in Case 5 above was taken, an eagle was observed and recorded on video attempting to catch foxes in enclosure 1. Since the time‐lapse cameras only take images every 10 min, it is likely that the actual interval was considerably less than 10 min.

A new video‐recording unit was installed in September 2021. This allows the recording of video onto disk when motion sensors are activated. In this specific case, two Arctic foxes, one white and one blue, are seen sprinting from the left‐hand side of the screen toward their den entrance. The eagle approaches the foxes at speed and is forced to end the pursuit as it descends and gets close to the structure erected immediately above the den entrance. The mitigation measure thus had the desired effect in this instance. Importantly, the rapidly approaching eagle was also able to detect the structure and avoid a potential collision (Figure A18).

The short video of the chase can be viewed online: https://youtu.be/ZAoJkqvpPzY

The eagle presumably moved toward enclosure 4 immediately thereafter and managed to kill a fox lying outside of an unprotected artificial den (Case 5, above).

### A.13 Bamboo sticks

Since we were unable to build structures over all dens, we added bamboo sticks to deter and create obstacles for approaching eagles that may attempt to swoop down and kill a fox (Figure A19). These are, however, difficult to deploy during the start of winter when there is little snow on the ground, yet represent a high‐risk period. In future, attempts will be made to drill holes in the ground during early winter. These can then be supplemented with additional sticks as snow depth increases.
